# Colon-Flushing in Typhoid Fever

**Published:** 1889-11

**Authors:** A. P. Buchman

**Affiliations:** Fort Wayne, Ind.


### COLON-FLUSHING IN TYPHOID FEVER.

BY A. P. BUCHMAN, M. D., FORT WAYNE, IND.

If, in a considerable number of cases of typhoid fever, who all suffer from the same fundamental lesions, present nearly, if not quite, a typical temperature-curve, and altogether run about the same course, and, including environments, receive the same treatment, one observes an amelioration of symptoms that can be safely credited to the *particular* course of treatment instituted and kept up all through the time of high-temperature manifestations, and, in a modified degree, during the period of convalescence, it will, I hope, serve the purpose for which this paper was written, viz., to call attention to the mode of treatment, to present the history of one of the cases.

On July 19th I was asked to prescribe for Mr. W——, aged about fifty, by his wife, she describing a case of simple intermittent malarial fever. I sent him a prescription composed chiefly of quinine, and heard nothing more of the patient until the evening of July 22d, a little more than three days having elapsed, when word was sent for me to call and see the patient. I saw him at 8:30 p. m., and found a temperature of 105° F., tongue red at tip and edges, centre dry and shining, some gurgling in the iliac fossæ, slight tympanites, and some half-dozen thin and rather offensive bowel-discharges during the day just passed; a general tired feeling, some headache, and such other symptoms as go to make up the typical typhoid condition. In addition to this there was acute mental worry, owing to unpleasant business complications. After prescribing and leaving general directions for the night, I left the house in no very pleasant frame of mind. Very acute, almost stinging mental worry, and a typhoid fever under headway for five days, with the subject working at his desk and on his feet all of this time, was, to say the least, a very disagreeable foreshadowing. I called again next morning, at nine o'clock, when the temperature was 103.5° F., tympanites much increased, some delirium, and very offensive bowel-discharges. This day was passed with extreme restlessness, and at my evening visit the temperature had gone up to 105.8° F., small, thready pulse, very rapid, bowels greatly dis tended with gas, and so much delirium that it was necessary for some one to remain at his bedside constantly. Just what to do next was a problem that presented itself for immediate solution.

I concluded to wash out the colon with a large enough quantity of water to thoroughly cleanse it, and to this end procured a fountain-syr inge of two-quart capacity; filling the reservoir with water directly from the hydrant, to which I added two ounces of listerine, I proceeded to fill the colon.

After waiting a few minutes, the patient was assisted on the commode; the entire contents of the colon were easily passed, thus relieving the ilio-colic valve, and permitting the accumulated gas in the small intestines to pass away as well. The odor accompanying this discharge was simply vile. The patient was now returned to the bed, and I passed my hand over the abdomen to note the condition. I found it flat and soft, all of the distension gone. Within the succeeding forty minutes his temperature had dropped to 101.4° F., the skin became moist, and before I left the house he had fallen into a quiet, peaceful sleep, which lasted, with one or two slight interruptions, the whole night. At this point I dropped all drug medication, save a one grain dose of quinine, three times a day, and substituted colon-flushing. Every time the temperature approached 103° F., the colon was thoroughly flushed, which was from one to three times every twenty-four hours. From July 26th to August 8th there was seemingly gradual but steady approach to the health-level. I say seemingly, for on the morning of August 9th at four o'clock, I was hastily summoned to the patient. The messenger stated that for an hour previous there seemed to be a great change for the worse. On arriving at the bedside I found the bowels rather distended, the breathing labored and slow—abdominal. My patient was in a state of shock nearly profound, resulting from, as I believed, intestinal hemorrhage. Until 3 a. m. everything had been quiet and favorable. Twice during the night he had taken nourishment with evident relish, but suddenly at this hour the whole scene changed, and they thought the patient dying. I immediately washed out the colon with hot water, first, for its stimulating effect, and, second, to relieve the bowels, as no blood had been discharged. Very soon the water, very bloody, came away, and soon I had the satisfaction of seeing the patient rally, and in less than two hours he seemed as well as he had been at any time the preceding day. From now on he convalesced rapidly. The colon-flushing was continued unremittingly, from one to three times every twenty-four hours, until the period of convalescence had ended, which was, to me, a surprisingly short one. I saw the patient last on the morning of August 20th, when he was moving about the room, and was gathering strength rapidly.

On July 20th I began to use the same method of treatment on a series of five other cases of typhoid fever, giving no drug save one or two-grain doses of salicylate of quinine three times a day. They have all progressed very favorably, and at this time (August 27th) I have so treated a total of thirteen cases. I wish to add here that at no time did I neglect to have a cold sponge-bath given at least once every twenty-four hours. The diet was chiefly milk.

In summing up, I find the situation about as follows:

1. That from one to three quarts of cold water can be easily and safely passed into the colon, which will rapidly lower a high temperature.

2. That I believe, in some of the cases, the water passed the ilio-colic valve, entering the small gut.

3. That tympanitic distention will always disappear with passing away of the water so injected.

4. That putrefactive fermentation of the bowel contents is prevented by such use of water.

5. That toxic substances are more rapidly absorbed by the cæcum than by any other portion of the intestinal canal, and that, by a judicious and careful washing with antiseptic water, we can prevent the absorption of such toxic substances, and prevent and modify general systemic poisoning.—*N. Y. Med. Record.*

